# Critical Load Prediction in Notched E/Glass–Epoxy-Laminated Composites Using the Virtual Isotropic Material Concept Combined with the Average Strain Energy Density Criterion

**DOI:** 10.3390/polym13071057

**Published:** 2021-03-27

**Authors:** Marcos Sánchez, Sergio Cicero, Ali Reza Torabi, Majid Reza Ayatollahi

**Affiliations:** 1 LADICIM (Laboratory of Materials Science and Engineering), University of Cantabria, E.T.S. de Ingenieros de Caminos, Canales y Puertos, Av/Los Castros 44, 39005 Santander, Spain; marcos.sanchez@unican.es; 2 Fracture Research Laboratory, Faculty of New Science and Technologies, University of Tehran, Tehran 14395-1561, Iran; 3 Fatigue and Fracture Research Laboratory, Center of Excellence in Experimental Solid Mechanics and Dynamics, School of Mechanical Engineering, Iran University of Science and Technology, Narmak, Tehran 16846, Iran; m.ayat@iust.ac.ir

**Keywords:** fracture, Virtual Isotropic Material Concept (VIMC), Average Strain Energy Density criterion (ASED), notch, laminated composite

## Abstract

This paper attempts to validate the application of the Virtual Isotropic Material Concept (VIMC) in combination with the average strain energy density (ASED) criterion to predict the critical load in notched laminated composites. This methodology was applied to E/glass–epoxy-laminated composites containing U-notches. For this purpose, a series of fracture test data recently published in the literature on specimens with different notch tip radii, lay-up configurations, and a number of plies were employed. It was shown that the VIMC–ASED combined approach provided satisfactory predictions of the last-ply failure (LPF) loads (i.e., critical loads).

## 1. Introduction

Within the field of engineering materials, composites have achieved significant prominence in the last few decades, its strength-to-weight ratio being one of the most relevant characteristics, so that it has become a perfect material in industries such as aerospace and automotive. A composite can be defined as that material resulting from the mixing of two or more constituent materials with significantly different properties (physical or chemical), which remain separate and distinct on a macroscopic level once the composite is formed, and generate a (composite) material with superior performance [1].

Among the different types of composites, continuous fibre-reinforced laminated composites stand out. They offer not only a high strength-to-weight ratio but may also have exceptional properties such as high durability, stiffness, flexural strength, and resistance to corrosion, wear, impact, and fire, among others. These composites have (generally) orthotropic mechanical properties, which, on the one hand, tend to present the best overall performance (compared to other types of composites) but, on the other hand, may deal with unique failure mechanisms that may be difficult to analyse (e.g., delamination or micro buckling). In any case, they are being used more and more as an alternative to other conventional materials, even for primary structural purposes [2].

Moreover, in many structural applications, components can be designed with different types of stress raisers (e.g., holes, notches, cut-outs), generating areas that are prone to crack initiation, and with the consequent loss of load-bearing capacity. Stress risers may also appear due to fabrication defects or operation damage. In any case, being able to predict accurately the load-bearing capacity (e.g., last-ply failure load) of laminated composites containing stress risers is essential for structural integrity purposes.

Conventionally, structural integrity assessment procedures or methodologies (e.g., [3,4,5]) address the analysis of crack-type defects. If they are directly applied to components containing notch-type defects, assuming that this type of defect behaves as cracks, they provide (mostly) over conservative results, due to the well-known notch effect (e.g., [6,7,8,9]). Thus, a great deal of research has been done to find specific methodologies for the assessment of notches. The different fracture theories that can be found in the literature dealing with the notch effect can be grouped into three different categories: (a) the global criterion [10,11,12], based on linear-elastic notch fracture mechanics, establishes that fracture occurs when the notch stress intensity factor reaches a critical value; (b) the local criteria, which bring together a series of approaches (e.g., the theory of critical distances (TDC) or the average strain energy density (ASED)) that have in common the analysis of the stress, strain, or energy fields at the defect tip. This paper focused, precisely, on the ASED criterion, which was validated in a wide range of materials [13,14,15]; (c) finally, the progressive damage models [16,17,18], which consider the material damage during the entire loading process, and the consequent changes in the stress distribution. The main issue found in the application of any of these failure models in laminated composites was to consider their orthotropic behaviour, the ply-by-ply analysis, and the first ply failure predictions, resulting in complex and time-consuming processes.

Recently, Torabi and Pirhadi developed a novel method to simplify the analysis of laminated composites, the so-called Virtual Isotropic Material Concept (VIMC). The authors successfully applied the VIMC on glass/epoxy-notched laminated composites containing U- and V-notches, in combination with two well-known brittle stress criteria: the maximum tangential stress (MTS) criterion and the mean stress (MS) criterion, to predict the last-ply failure (LPF) loads under both mode I and mixed-mode I/II loadings [19,20,21,22,23].

With all this, and based on the experimental results published by Torabi and Pirhadi in [19], this paper attempted to expand the use of VIMC to analyse U-notched laminated composites in combination with the energetic ASED criterion. Thus, Section 2 provides the theoretical framework of the work, including a description of both the VIMC and the ASED criterion. Section 3 presents the materials and methods used for the prediction of LPF load of U-notched laminated composites. Section 4 gathers the experimental results and provides the LPF load predictions provided by the proposed methodology, together with the corresponding discussion. Finally, Section 5 presents the main conclusions.

## 2. Theoretical Background

### 2.1. The ASED Criterion

**Figure 1:**
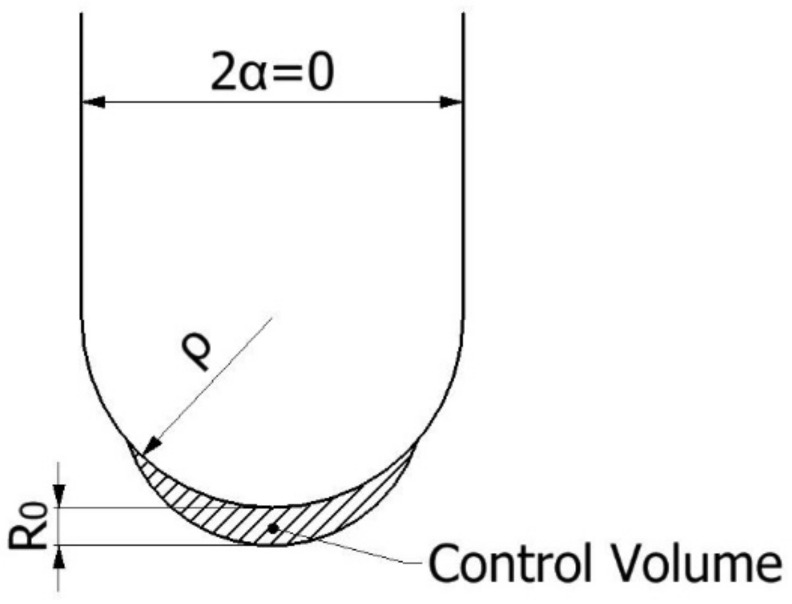
Control volume (area) for U-notch (2α = 0) under mode I loading.

The average strain density energy (ASED) criterion, whose fundamentals were developed by Shi [24], assumes that, under mode I loading, fracture occurs when the average value of the strain energy density ($\bar{W}$) over a certain control volume (defined by radius R_0_, see Figure 1) reaches a critical value (1) [25]:

(1) $$\bar{W}=W_{c}$$

When the material exhibits linear-elastic behaviour, W_c_ is directly provided by Equation (2), as proposed in [25].

(2) $$W_{c}=\frac{\sigma_{u}^{2}}{2E}$$

σ_u_ being the ultimate tensile strength and E being Young’s modulus.

When the notch opening angle is zero (2α = 0, see Figure 1), as occurs in the case of cracks or U-notch, R_0_ can be defined in terms of the fracture toughness (K_c_), the ultimate tensile strength (σ_u_), and the Poisson’s ratio (υ). If the material is operating under plane strain conditions, R_0_ is expressed by Equation (3), while under plane stress conditions, R_0_ is given by Equation (4).

(3) $$R_{0}=\frac{\left(1+v\right)\left(5-8v\right)}{4\pi }{\left(\frac{K_{c}}{\sigma_{u}}\right)}^{2}$$

(4) $$R_{0}=\frac{\left(5-3v\right)}{4\pi }{\left(\frac{K_{c}}{\sigma_{u}}\right)}^{2}$$

Those situations between plane strain and plane stress conditions require interpolation between Equations (3) and (4) to define R_0_. In this sense, Equation (5) indicates the upper limit of fracture resistance where plane strain conditions may be considered to be dominant [6]:

(5) $$K_{c}=\sigma_{y}{\left(\frac{B}{2.5}\right)}^{1/2}$$

Analogously, the onset of plane stress conditions is attained when the fracture resistance exceeds the value given by Equation (6) [6]:

(6) $$K_{c}=\sigma_{y}{\left(\pi B\right)}^{1/2}$$

σ_y_ being the yield or proof strength, and B being the specimen thickness. For those situations in which the fracture resistance value is located within these two limits, R_0_ may be obtained by interpolation between Equations (3) and (4).

Once R_0_ is known, the ASED criterion requires the average strain energy density ($\bar{W}$) to be derived within the corresponding control volume. Lazzarin and Berto [9] derived a useful, straightforward analytical expression, gathered in Equation (7):

(7) $$\bar{W}=F\left(2\alpha \right)H\left(2\alpha ,\frac{R_{0}}{\rho }\right)\frac{\sigma_{\max}^{2}}{E}$$

where F is a function that depends on the notch opening angle (2α) and whose values are gathered in [9]. For this work, it sufficed to say that for U-shaped notches (2α = 0°) F is equal to 0.785. Furthermore, H is another function depending on notch geometry (opening angle and notch radius) and material properties (R_0_ and, thus, fracture toughness, ultimate tensile strength, and Poisson´s ratio), whose values (for U-notch type defect) may easily be obtained from Table 1 [9]. Finally, σ_max_ is the maximum stress at the notch tip at fracture conditions.

With all these Equations (1)–(7), it is straightforward to assess notched components according to the ASED fracture criterion. The results shown in the literature reveal the accuracy of this approach as long as the material being analysed presents a linear-elastic behaviour (e.g., [9,13,14,15])

### 2.2. The Virtual Isotropic Material Concept

The main assumption of the VIMC is that it equates a real laminated composite with orthotropic behaviour to a virtual brittle plate of the same geometry with isotropic behaviour [21]. Once this is done, if the VIMC is correct, well-known fracture methodologies (e.g., TCD, ASED) can be directly applied.

A review of the literature dealing with the fracture analysis of engineering materials containing notches revealed that there are two essential material parameters: the characteristic strength (σ_f_) and the fracture toughness (K_c_). The characteristic strength is also referred to as the critical stress or the inherent strength, depending on the approach, and, assuming fully linear-elastic conditions, is generally assumed to be equal to the material ultimate tensile strength (e.g., see Equations (2)–(4). The VIMC, at the same time, requires (just) two important properties of the laminated composite to be defined, namely the ultimate tensile strength (σ_u_) and the trans-laminar fracture toughness (K_TL_). The correct definition of these two properties is the main issue of the application of the VIMC. As soon as they are known, they are considered as the K_c_ and the σ_f_ of the virtual isotropic material, and the notch assessment (in terms of LPF load predictions) of the laminated composite being analysed follows the same methodologies used for isotropic materials. Thus, it should be noted that, when using the VIMC, it is not required to perform a large experimental program to determine the mechanical properties in different directions, such as longitudinal Young´s modulus (E_x_), lateral Young´s modulus (E_y_), in-plane Poisson´s ratio (υ_xy_), or shear elastic modulus (G_xy_).

Summarising, once the material characterisation difficulty has been overcome, the application of any of the well-known brittle fracture criteria (e.g., ASED criterion) can go ahead simply by using the obtained values of σ_u_ and K_TL_ as σ_f_ and K_c_, respectively. The VIMC is, then, a relevant tool to facilitate the prediction of the critical loads of brittle or quasi-brittle laminated composites (e.g., E/glass–epoxy-laminated composites).

## 3. Materials and Methods

The VIMC–ASED criterion was applied here to fibre-reinforced laminated E/glass–epoxy composites. The entire experimental program was previously conducted by Torabi and Pirhadi, whose results were published in [21], where the reader may find additional details to those gathered here.

The laminated composites were manufactured using E-glass fibre (between 56% and 59% of fibre volume), epoxy resin (Epon 828), and Siclo-Aliphatic-Amin hardener. The resulting composite plates were fabricated using the vacuum bag-autoclave moulding technique, including bleeders located on the top and bottom surfaces. In order to validate the VIMC–ASED methodology independently of the composite structure, layers with three different fibre orientations (unidirectional (0)_s_, cross-ply (0/90/0/90)_s_, and quasi-isotropic (0/90/±45)_s_ and two different numbers of layers (8-ply and 16-ply) were utilised. These generated 6 different material conditions. The thickness of each lamina was approximately 0.28 mm, with the total thickness of each laminate configuration being between 2.9 mm and 3.1 mm for 8 layers, and between 5.6 mm and 5.8 mm for the 16-layer specimens. With these different thicknesses, it was possible to determine whether the VIMC–ASED criterion proposed in this work was independent of the number of layers.

**Figure 2:**
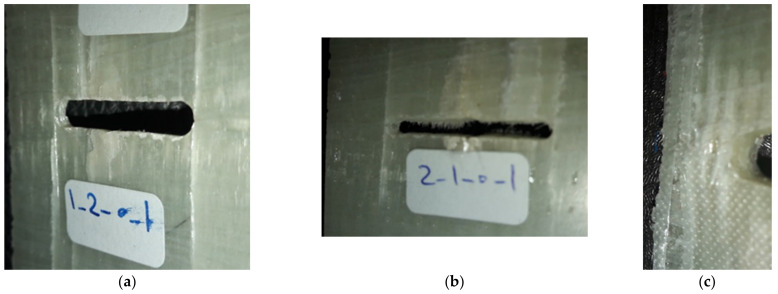
Some of the U-notched composite samples after last-ply failure (LPF). (**a**) Unidirectional, (**b**) cross-ply, and (**c**) quasi-isotropic configurations.

The experimental program encompasses three different types of tests. Two of them (characterisation tests) were completed for the material characterisation (σ_u_ and K_TL_) itself, which was subsequently used as an input for the VIMC–ASED analysis. The last type (validation tests) consisted of fracture tests of laminated composites weakened by U-notches in order to determine the LPF loads and compare them with the VIMC–ASED predictions. Figure 2 shows some of the U-notched composite samples after LPF.

In the unidirectional composite (Figure 2a), local damage first nucleated from the notch tip, then grew slowly during loading, and, finally, it reached the long fibres that were oriented perpendicular to the damage growth direction. As a result, at the onset of LPF, a long crack parallel to the fibres was recognised in the U-notch neighbourhood. In Figure 2b,c, corresponding to the cross-ply and quasi-isotropic lay-ups, different patterns were observed at LPF onset. The main difference between the failure pattern of the unidirectional configuration with the patterns of the two other configurations was that in the unidirectional composite, the pattern was mainly longitudinal and no lateral pattern could be recognised, whereas, in the two latter cases, failure patterns were both longitudinal and lateral due to the presence of 90° plies in the lay-up.

As explained before, only two parameters are necessary to completely describe the VIMC, namely the ultimate tensile strength (σ_u_) and the trans-laminar fracture toughness (K_TL_) of the real laminated composite. σ_u_ was obtained, for each laminated composite, through a tensile test at a loading rate of 2 mm/min, utilising unnotched samples, following the test procedure and the requirements of sample geometry specified in the standard ASTM D3039 [26]. Additionally, the bulk value of E was calculated by the same tensile test. Moreover, the K_TL_ parameter was determined by fracture tests on pre-cracked samples according to the standard ASTM E1922 [27]. The single-edge narrow notches (i.e., the pre-cracks) in the compact-tension (CT)-like specimens suggested by ASTM E1922 [27] were introduced by using a diamond wheel cutter with a thickness of 0.3 mm, fulfilling the standard geometrical conditions (see [21] for details). In both tensile and fracture characterisation tests, three specimens were tested per lay-up configuration.

**Figure 3:**
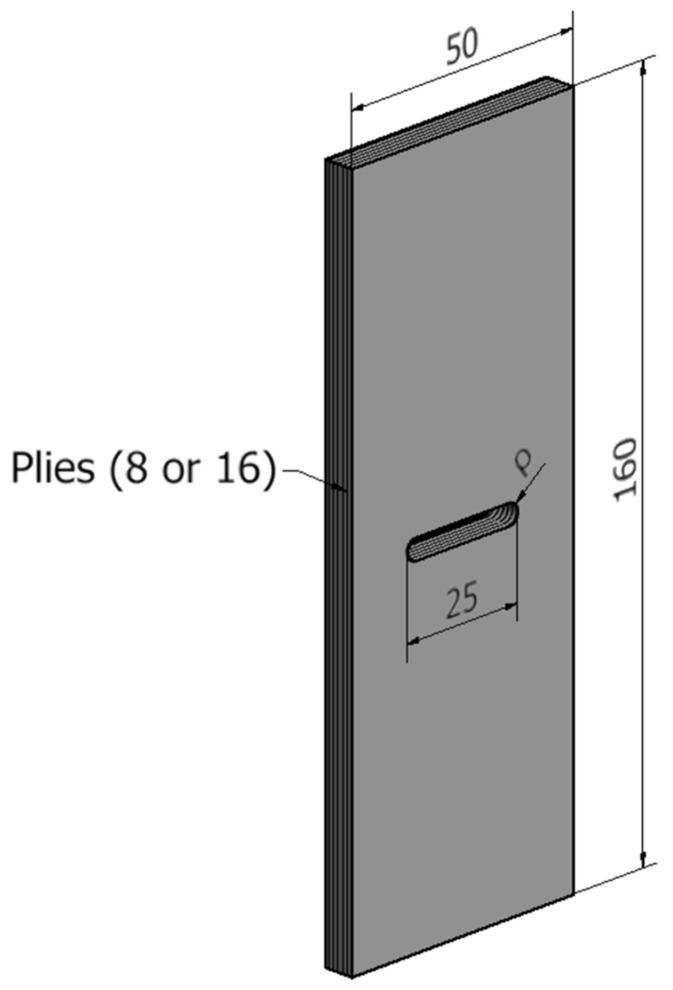
Schematic of the U-notched laminated composite specimen. All dimensions are in mm. The lay-up configurations are (0)_s_, (0/90/0/90)_s_, and (0/90/±45)_s_.

After characterisation tests, validation tests were completed. The objective was to complete the experimental tests on the U-notched specimens and to compare the critical load (LPF load) with the predictions provided by the VIMC–ASED combined criterion. The main dimensions of the notched specimens are shown in Figure 3, with ρ being the notch tip radius and taking values of 1, 2, and 4 mm. Again, further details may be found in [21].

Once the whole experimental program was completed, the predictions of critical (LPF) loads in E/glass–epoxy-laminated composites using the VIMC–ASED criterion were derived. As seen above, the application of the ASED criterion depends on several material properties, such as the fracture toughness, the ultimate tensile strength, Young’s modulus, and the Poisson´s ratio. Whereas the three first parameters were directly derived from the experimental program (tensile and fracture tests), the Poisson´s ratio was not determined experimentally in the original tests [21]. Consequently, this parameter was selected from the literature, and this was accompanied by a sensitivity analysis on the influence of the Poisson´s ratio being selected in the final LPF load predictions.

Considering the mechanical properties provided by the VICM, it was straightforward to calculate the critical strain energy (W_c_; Equation (2)). This W_c_ must be compared with the average strain energy density ($\bar{W}$) within the control volume (R_0_), a process requiring the following steps:

- Determine R_0_, considering the material properties of the VIMC. R_0_ follows Equation (3) or (4), depending on the plane strain vs. plane stress conditions. Equations (5) and (6) allowed the limits for both conditions to be estimated, and, in case of intermediate situations, linear interpolation may be used to derive R_0_.
- The F function (see Equation (7)) was assumed to be equal to 0.785, given that in all the tested notched specimens 2α = 0.
- The value of the H function was derived from Table 1 for each material and notch radius.
- The maximum stress (*σ*_max,VIMC–ASED_) at the notch tip was the only unknown in Equation (7), so it may be directly derived for each material and notch radius. Here, it should be noted that *σ*_max,VIMC–ASED_ corresponded to the stress state at critical conditions (i.e., when the LPF load is applied).

**Figure 4:**
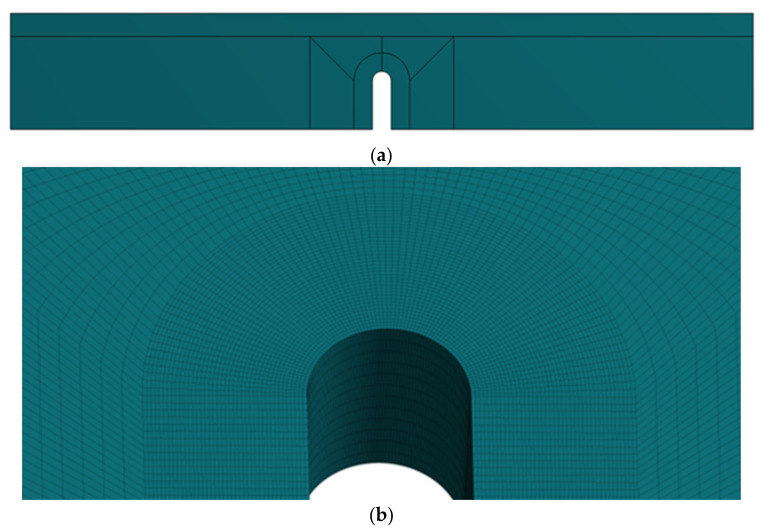
(**a**) Structured mesh used for the 3D model; (**b**) detail of the mesh near the notch tip.

Finally, in order to obtain the critical loads provided by the VIMC–ASED criterion (P_VIMC–ASED_), the notched fracture specimens were modelled and analysed by finite element analysis (FEA). The simulations were performed in linear-elastic conditions using the finite element software ANSYS 19.2 (Ansys Inc, Canonsburg, PA, USA). The three-dimensional models were partitioned, obtaining a structured mesh composed of 20-node hexahedron elements (see Figure 4a). Additionally, the mesh around the notch tip was refined (minimum element about 0.03 mm), making sure to correctly capture the high-stress gradients generated by the notches (see Figure 4b). The simulation was performed with half of the model taking advantage of the symmetry (although one-quarter of the specimens would have been enough, the computational effort of considering one half was actually very limited). A unit load was applied at the top face (0.5 N in the half being considered), whereas the bottom face was fixed and the nodes belonging to the symmetric face were restrained in the perpendicular direction of the symmetry plane. With all this, the maximum principal stress in the notch root was obtained for the unit load (σ_max,UL_). The predictions of P_VIMC–ASED_ were obtained by linearly scaling the values obtained from the FEA until the maximum stress values provided by VICM–ASED criterion are reached:

(8) $$P_{\mathrm{VIMC}-\mathrm{ASED}}=\sigma_{\max,\;\mathrm{VIMC}-\mathrm{ASED}}/\sigma_{\max,\mathrm{UL}}$$

## 4. Results and Discussion

The mechanical properties (average values) obtained in the experimental characterisation program are gathered in Table 2 [21]. These properties, obtained from conventional tensile and fracture tests, were the only required inputs of the VIMC. It may be observed that there were slight effects of the number of plies and significant effects of the fibre orientation. 16-ply materials had a tendency to generate slightly higher ultimate tensile strength and Young´s modulus than 8-ply materials, which on the contrary tended to develop higher values of K_TL_. This latter observation may be more related to the different conditions of plane stress/plane strain between the two different ply numbers. Concerning the fibre orientation, unidirectional orientation developed higher material properties (both tensile and fracture), whereas cross-ply and quasi-isotropic orientation presented less significant differences between them. Unidirectional specimens (0) had the best material properties as a result of the relation between the stress state and fibre direction, given that the fibres were precisely oriented in the same direction as the tensile stresses causing the fracture process.

The experimental results of the validation fracture tests (critical or LPF loads) for the three lay-up configurations are shown in Table 3. Similarly to fracture characterisation tests, it could be observed how the unidirectional lay-up configuration developed higher critical loads, but some further observations may be made:

- The Cross-ply lay-up configuration, which presents the lowest values of K_TL_, also developed the lowest critical loads in the validation fracture tests.
- The critical loads for the 16-ply specimens were not double those obtained for 8-ply samples. These agreed with the higher fracture toughness observed in thinner specimens, much closer to plane stress conditions.
- The notch effect (i.e., increase in critical load with the notch tip radius) existed in all conditions, but it was not very pronounced. Fracture loads in specimens with a notch radius of 1 mm were approximately 15–20% lower than those developed by specimens with a notch radius of 4 mm.
- Most of the notch effects were observed when comparing the critical loads of specimens with notch radii of 1 mm and 2 mm. The differences observed between specimens with notch radii of 2 mm and 4 mm were significantly less.

**Table 3:** Experimental critical loads of U-notched specimens for each lay-up configuration [21].

Lay-Up Configuration	*ρ* (mm)	Number of Layers	P_Exp_ (kN)
Unidirectional (0)	1	8-ply	23.70
	2		26.30
	4		27.40
	1	16-ply	41.70
	2		43.90
	4		44.20
Cross-ply (0/90/0/90)	1	8-ply	14.30
	2		16.90
	4		17.20
	1	16-ply	26.80
	2		29.10
	4		29.85
Quasi-isotropic (0/90/0/±45)	1	8-ply	16.50
	2		18.30
	4		18.90
	1	16-ply	26.50
	2		29.80
	4		30.90

Table 4 gathers the VIMC–ASED predictions for the critical loads (P_VIMC–ASED_), together with the different inputs of the analysis. The material properties used in the analysis were those gathered in Table 2, and the Poisson´s ratio considered here was 0.2, according to the literature [28,29]. In any case, a sensitivity analysis was performed to check how the Poisson´s ratio affected the final predictions, revealing that results were not particularly sensitive to the specific value used in this parameter (e.g., considering a Poisson’s ratio of 0.25 generated variation of approximately 0.04% in P_VIMC–ASED_ predictions). Finally, it is also important to mention that, in most cases, the values of K_TL_ (see Table 2) were found between the plane strain limit (Equation (5)) and the plane stress onset (Equation (6) and, thus, R_0_ was obtained by linear interpolation between Equations (3) and (4).

**Figure 5:**
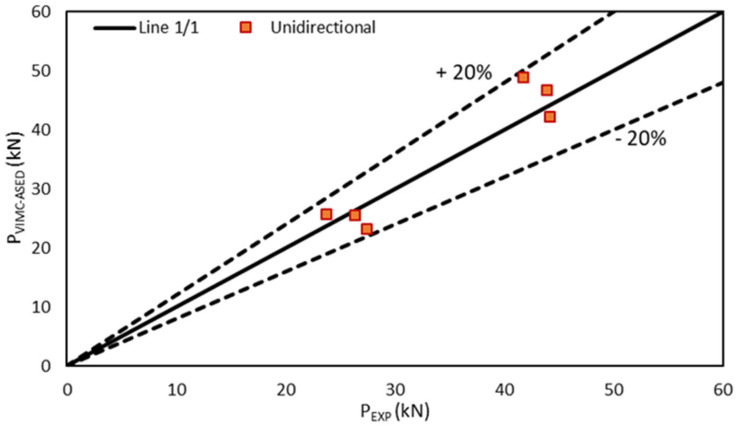
Comparison between the critical load prediction and experimental results in unidirectional specimens (0).

**Figure 6:**
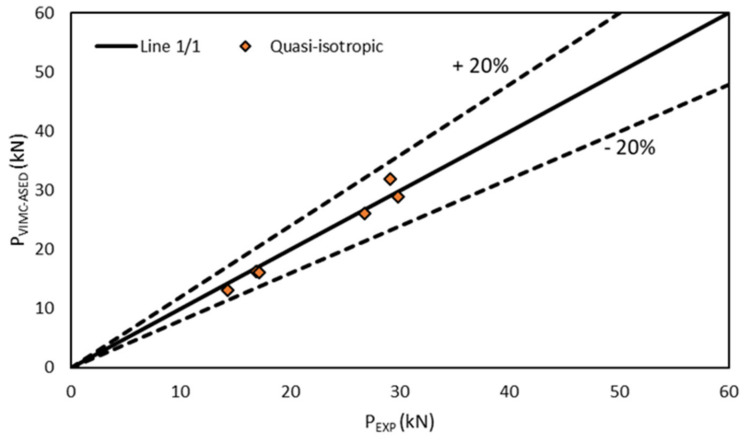
Comparison between the critical load prediction and experimental results in quasi-isotropic specimens (0/90/±45).

**Figure 7:**
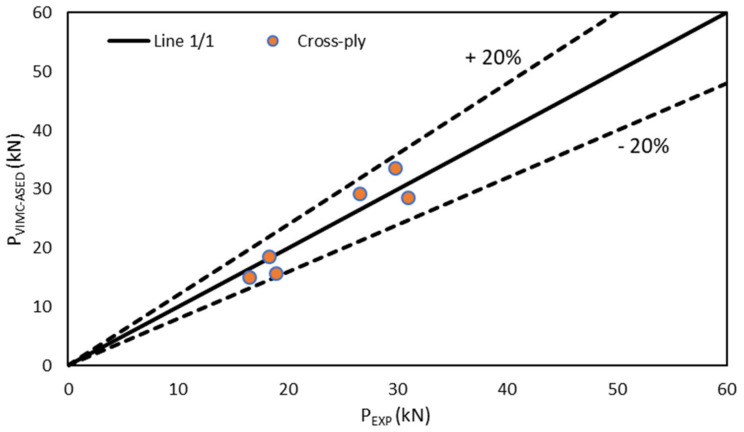
Comparison between the critical load prediction and experimental results in cross-ply specimens (0/90/0/90).

Figure 5, Figure 6 and Figure 7 compare the experimental results with the corresponding VIMC–ASED predictions, revealing good accuracy for the different lay-up configurations. Further, all the estimated values were within the scatter of ±20% accepted in the fracture mechanics research field [6,7,8,9]. The average deviation from the experimental results was +0.4% for the unidirectional composite, −2.4% for the cross-ply composite, and −2.7% in the case of the quasi-isotropic composite, the highest deviation being −18%, −8.6%, and −19.8% for unidirectional, cross-ply, and quasi-isotropic lay-ups, respectively. Moreover, the accuracy was adequate for both 8-ply and 16-ply materials, although there was a tendency to provide conservative predictions (P_VIMC–ASED_ < P_experimental_) for 8-ply materials and slightly non-conservative predictions in the case of 16-ply materials.

It may also be observed that the predictions often generated lower critical loads when the notch tip radius increased. This was caused by the interpolation process when obtaining R_0_ and, although this was contrary to empirical observations, it provided a correction based on the plane stress vs. plane strain situations which, overall, improved the general accuracy of the predictions.

## 5. Conclusions

This paper proposed and validated a methodology for the prediction of critical loads (or LPF loads) in E/glass–epoxy laminated composites weakened by U-notches. The methodology was based on the combined use of the Virtual Isotropic Material Concept and the average strain energy density criterion.

The VIMC allowed the material’s mechanical properties on laminated composites to be sufficiently defined by simply testing both tensile and pre-cracked fracture specimens, obtaining the corresponding ultimate tensile strength (σ_u_), and trans-laminar fracture toughness (K_TL_), respectively. The real laminated composite material was then substituted by a virtual isotropic one, whose analysis was performed as it is usually done in isotropic materials. This procedure was completed in this work for different lay-up configurations (unidirectional, cross-ply, and quasi-isotropic) and numbers of plies. Then, for the same lay-up configurations and number of plies, validation U-notched specimens with three different notch radii were tested to determine the corresponding experimental critical loads, which were finally compared with the predictions provided by the VIMC–ASED combined criterion.

The VIMC–ASED combined criterion provided accurate predictions of the critical loads for the different lay-up configurations and a number of plies. This allowed the fracture loads of U-notched composite laminates to be simply estimated by using conventional approaches used in isotropic materials.

## Figures and Tables

**Table 1:** Values of H for U-shaped notches [9].

R_0_/*ρ*	H				
	ν = 0.1	ν = 0.15	ν = 0.2	ν = 0.25	ν = 0.3
0.0005	0.6294	0.6215	0.6104	0.596	0.5785
0.001	0.6286	0.6207	0.6095	0.5952	0.5777
0.005	0.6225	0.6033	0.6033	0.5889	0.5714
0.01	0.6149	0.6068	0.5956	0.5813	0.5638
0.05	0.5599	0.5515	0.5401	0.5258	0.5086
0.1	0.5028	0.4942	0.4828	0.4687	0.4518
0.3	0.3528	0.3445	0.3341	0.3216	0.3069
0.5	0.2672	0.2599	0.2508	0.2401	0.2276
1	0.159	0.1537	0.1473	0.1399	0.1314

**Table 2:** Mechanical properties of the different ply configurations employed [21].

Material Property	Unidirectional		Cross-Ply		Quasi-Isotropic	
	8-Ply	16-Ply	8-Ply	16-Ply	8-Ply	16-Ply
σ_u_ (MPa)	858 ± 7.0	876 ± 4.0	489 ± 8.2	498 ± 4.4	425 ± 4.4	442 ± 5.3
K_TL_ (MPa∙m^1/2^)	51.2 ± 1.2	47.8 ± 1.3	39.2 ± 1.2	36.5 ± 2.7	42.6 ± 1.9	40.2 ± 5.3
E (GPa)	45.2	46	30.6	31.1	33.2	34

**Table 4:** Average strain energy density (ASED) parameters together with the Virtual Isotropic Material Concept (VIMC)–ASED critical loads.

Lay-up Configuration	*ρ* (mm)	Number of Layers	R_0_ (mm)	R_0_/*ρ*	H	W_c_ (MPa)	σ_max_ (MPa)	P_VICM-ASED_ (kN)
Unidirectional (0)	1.00	8-ply	1.19	1.19	0.15	8.14	1784.17	25.70
	2.00	8-ply	1.19	0.60	0.23	8.14	1425.11	25.55
	4.00	8-ply	1.19	0.30	0.34	8.14	1182.21	23.22
	1.00	16-ply	0.97	0.97	0.15	8.34	1787.65	48.86
	2.00	16-ply	0.97	0.49	0.26	8.34	1380.47	46.67
	4.00	16-ply	0.97	0.24	0.38	8.34	1139.62	42.11
Cross-ply (0/90/0/90)	1.00	8-ply	3.52	3.52	0.15	2.72	883.77	13.17
	2.00	8-ply	3.52	1.76	0.15	2.72	883.77	16.19
	4.00	8-ply	3.52	0.88	0.17	2.72	817.26	16.19
	1.00	16-ply	2.79	2.79	0.15	2.87	919.12	26.02
	2.00	16-ply	2.79	1.40	0.15	2.87	919.12	31.99
	4.00	16-ply	2.79	0.70	0.21	2.87	770.06	28.98
Quasi-isotropic (0/90/±45)	1.00	8-ply	2.21	2.21	0.15	3.91	1016.85	15.15
	2.00	8-ply	2.21	1.10	0.15	3.91	1016.85	18.62
	4.00	8-ply	2.21	0.55	0.24	3.91	796.37	15.77
	1.00	16-ply	1.78	1.78	0.15	3.99	1035.57	29.31
	2.00	16-ply	1.78	0.89	0.17	3.99	965.14	33.59
	4.00	16-ply	1.78	0.45	0.27	3.99	760.37	28.61

## Data Availability

The data presented in this study are available on request from the corresponding authors.
